# Laboratory-based surveillance of antimicrobial resistance in regions of Kenya: An assessment of capacities, practices, and barriers by means of multi-facility survey

**DOI:** 10.3389/fpubh.2022.1003178

**Published:** 2022-11-28

**Authors:** Rehema Moraa Moirongo, Leslie Mawuli Aglanu, Maike Lamshöft, Brian Omondi Adero, Solomon Yator, Stephen Anyona, Jürgen May, Eva Lorenz, Daniel Eibach

**Affiliations:** ^1^Department of Infectious Disease Epidemiology, Bernhard Nocht Institute for Tropical Medicine (BNITM), Hamburg, Germany; ^2^Global Health and Infectious Diseases Research Group, Kumasi Centre for Collaborative Research in Tropical Medicine, Kumasi, Ghana; ^3^Department of Internal Medicine/Infectious Diseases, University Medical Centre Groningen, University of Groningen, Groningen, Netherlands; ^4^German Center for Infection Research (DZIF), Braunschweig, Germany; ^5^Rangwe Sub County Hospital, Homa Bay, Kenya; ^6^Department of Biochemistry and Molecular Biology, University of Bremen, Bremen, Germany; ^7^Centre for Microbiology Research (KEMRI-CMR), Kenya Medical Research Institute, Nairobi, Kenya; ^8^Department of Tropical Medicine I, University Medical Center Hamburg-Eppendorf (UKE), Hamburg, Germany; ^9^Institute of Medical Biostatistics, Epidemiology and Informatics, University Medical Centre of the Johannes Gutenberg University Mainz, Mainz, Germany

**Keywords:** surveillance of antimicrobial resistance, antimicrobial susceptibility testing, quality assurance, laboratory infrastructure, Kenya

## Abstract

**Background:**

Adequate laboratory capacity is critical in the implementation of coherent surveillance for antimicrobial resistance (AMR). We describe capacities and deficiencies in laboratory infrastructure and AMR surveillance practices among health facilities in Kenya to support progress toward broader sustainable laboratory-based AMR surveillance.

**Methods:**

A convenience sample of health facilities from both public and private sectors across the country were selected. Information was obtained cross-sectionally between 5th October and 8th December 2020 through online surveys of laboratory managers. The assessment covered quality assurance, management and dissemination of AMR data, material and equipment, staffing, microbiology competency, biosafety and certification. A scoring scheme was developed for the evaluation and interpreted as (80% and above) facility is adequate (60–79%) requires some strengthening and (<60%) needing significant strengthening. Average scores were compared across facilities in public and private sectors, rural and urban settings, as well as national, county, and community levels.

**Results:**

Among the participating facilities (*n* = 219), the majority (*n* = 135, 61.6%) did not offer bacterial culture testing, 47 (21.5%) offered culture services only and 37 (16.9%) performed antimicrobial susceptibility testing (AST). The major gaps identified among AST facilities were poor access to laboratory information management technology (LIMT) (score: 45.9%) and low uptake of external quality assessment (EQA) programs for cultures (score 67.7%). Access to laboratory technology was more than two-fold higher in facilities in urban (58.6%) relative to rural (25.0%) areas. Whilst laboratories that lacked culture services were found to have significant infrastructural gaps (average score 59.4%), facilities that performed cultures only (average score: 83.6%) and AST (average score: 82.9%) recorded significantly high scores that were very similar across areas assessed. Lack of equipment was identified as the leading challenge to the implementation of susceptibility testing among 46.8% of laboratories.

**Conclusions:**

We identified key gaps in laboratory information management technology, external quality assurance and material and equipment among the surveyed health facilities in Kenya. Our findings suggest that by investing in equipment, facilities performing cultures can be successfully upgraded to provide additional antimicrobial susceptibility testing, presenting a chance for a major leap toward improved AMR diagnostics and surveillance in the country.

## Introduction

The growing public health threat of antimicrobial resistance (AMR) increasingly undermines our ability to treat and prevent infections caused by bacteria with existing antibiotic medication. AMR can be effectively minimized through coherent surveillance that facilitates continuous capture and onward sharing of reliable data for the development of targeted curtailing interventions on local, national, and global levels (1–3). Primarily, laboratory testing is the foundation for detecting resistance (4) and providing essential information for clinicians to institute appropriate treatment regimens for patients, thereby limiting potential misuse of drugs. However, where quality laboratory services are not always available, treatment often involves untargeted empirical administration of antimicrobials, including broad-spectrum agents, accelerating the development, and spread of drug resistant microorganisms.

In Kenya, AMR data are mainly generated by the Kenya Medical Research Institute (5), supplemented by central reference laboratories, large hospitals, and sentinel sites set up to address specific pathogens of major public health concern. Surveillance for AMR extends to facilities run by individuals or corporations, and in some cases externally funded research units. The past decade has seen a significant increase in effort to describe and tackle the burden due to drug-resistant infections in the country (6), although overall nationwide surveillance is still at the early stages with AMR data generally remaining patchy (7). Over the years, many studies have demonstrated variable resistance rates in microorganisms that are associated with unfavorable outcomes in hospital and community settings, such as those that cause among others; tuberculosis, meningitis, pneumonia, and gastrointestinal diseases (8–14). Findings from these studies and other initiatives fighting AMR highlight the need for horizontal (15–18) as well as vertical (19) strengthening of laboratory capacity to promote widespread detection of resistance and to create strong evidence for optimal AMR response.

Our study applies quantitative scores to assess health laboratories in Kenya to identify deficiencies in resources, infrastructural and operational capacities regarding dimensions of surveillance systems emphasized by the WHO strategy on the containment of AMR (20). Recognizing resource scarcity, this assessment could guide planning, prioritization, and implementation of project activities to support progress toward broader sustainable laboratory-based AMR surveillance in low-income settings.

## Methods

### Survey tool

**Figure 1:**
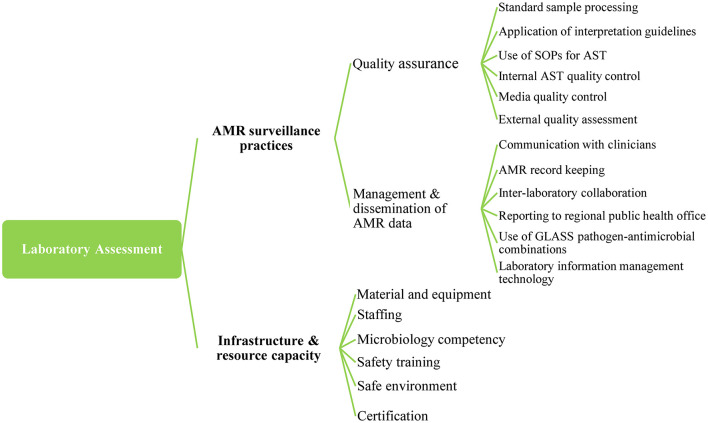
Dimensions, subdimensions, and indicators of tool developed for the assessment of health facilities in Kenya 2020. AMR, Antimicrobial Resistance; AST, Antimicrobial Susceptibility Testing; GLASS, Global Antimicrobial Resistance and Use Surveillance System; SOPs, Standard operating procedures.

We composed a detailed online survey based on the WHO Antimicrobial Resistance Surveillance Questionnaire for Assessment of National Networks (21) and the Stepwise Laboratory (Quality) Improvement Process Toward Accreditation (SLIPTA) checklist^1^. The survey combined two dimensions: (i) AMR surveillance practices and (ii) Laboratory infrastructure and resource capacity. Dimension 1, AMR surveillance practices, was further grouped into two subdimensions (quality assurance and management and dissemination of AMR data) of six indicators each. Dimension 2 combined six subdimensions with a variable number of indicators (Supplementary File 1). The areas addressed by the survey are summarized on Figure 1.

A scoring system for the indicators was designed, adapting previously established criteria (22–24). Each indicator was scored on a scale of 0–1 as follows: A “yes” or “present and functional” gave an index value of 1, “partial” or “other” or “present and non-functional” 0.5 and a “no” or “absent” 0. For the dimension “infrastructure and resource capacity,” indicators were reviewed and weighted based on their necessity for laboratory-based AMR surveillance. The weight values were assigned in indices and set from 0 to 1 as described in Supplementary File 1. All indicators of dimension “AMR surveillance practices” were weighted equally with value 1 as there are currently no standardized guidelines pertinent to evaluating the indicators. The weighting criterion was defined by an expert team of the department of Infectious Disease Epidemiology of the Bernhard Nocht Institute of Tropical Medicine and Kumasi Center for Collaborative Research. We piloted the questionnaire at a bacteriology laboratory in Germany before initiating assessment.

### Sampling and data collection

A combination of convenience and snowball sampling methods was used in the study, taking advantage of previously established in-country networks. Only laboratories with human health services were included in the assessment covering elements such as their level, affiliation, type and urbanicity. Facility level refers to the six hierarchical tiers of the Kenyan healthcare service delivery system (25). In the tier structure, the lower-level facilities including community units (level 1) and health dispensaries (level 2) are typically the first points of care for the management of minor ailments like common cold, uncomplicated malaria and diarrhea. On the other hand, county (level 5) and national (level 6) referral hospitals, handle more severe cases that require specialized care^2^. Facility affiliation relates to ownership i.e., public or private. In this study, non-public entities include those supported by faith-based and non-government organizations as well as those run for profit by private companies or individuals. We described facility type based on bacteriology activity, particularly the availability of culture services and antimicrobial susceptibility testing (AST). The study area was defined as either urban; densely populated regions with compact road networks, or rural; moderate to sparsely populated regions with poor road network. Information was obtained cross-sectionally between 5th October and 8th December 2020, through online surveys of laboratory managers responsible for AMR surveillance, microbiology, and laboratory systems.

### Data management and analysis

The data were collected and managed using REDCap electronic data capture tools hosted at the Bernhard Nocht Institute for Tropical Medicine, Germany^3^. Reconciliation of inconsistencies and missing data was done before conducting statistical analyses. The total scores of all the indicators, subdimensions, and dimensions were converted into percentages. The total indicator scores were obtained as averages of all the participating facilities indicators scores. For the dimension “AMR surveillance practices,” overall scores per indicator were calculated as average indicator scores of facilities with susceptibility testing, whereas subdimension scores were obtained as average indicator scores. Performance strengths and proportions of facilities across the AMR surveillance areas are displayed on a stacked bar chart. For dimension 2, laboratory infrastructure and resource capacity, we compared average subdimension scores for facilities without culture testing, those with cultures only and those undertaking antimicrobial susceptibility testing, stratifying by affiliation, urbanicity and level. Percentage values are interpreted as (80% and above) facility is adequate (60–79%) requires some strengthening (<60%) needing significant strengthening, as similarly applied in other studies (23, 24).

### Ethical considerations

The study was reviewed and approved by the National Commission for Science, Technology, and Innovation (NACOSTI) License No. NACOSTI/P/20/4083 and authorization to carry out the assessment granted by the Kenyan Ministry of Health (MoH). To ensure confidentiality, respondent identification information was only accessed by authorized people of the study team during analysis.

## Results

### Study facilities

**Figure 2:**
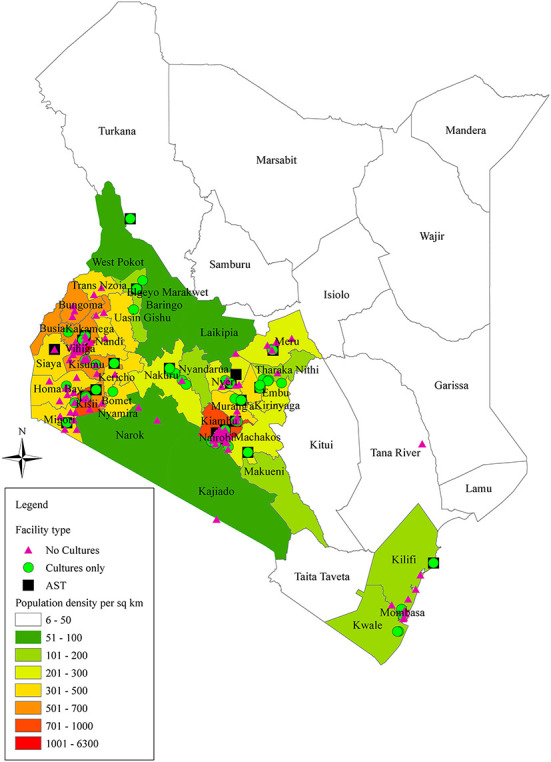
Map of Kenya representing the geographic locations of the health facilities completing the survey and the population density by location. The figure was generated using pandas and GeoPandas software libraries in Python. Facility type (No cultures; lacking culture testing, Cultures only; performing cultures but no antimicrobial susceptibility testing; AST, Antimicrobial susceptibility testing).

Between 5th October and 8th December 2020, 466 REDCap survey links were sent to health facilities across the country. A response rate of 73.2% (*n* = 341) was recorded at the end of the data collection period. Following cleaning and reconciliation of duplicates, incomplete and inconsistent forms, surveys from 219 (64.2%) of the submitted forms were considered for analysis. Most of the participating facilities are located in the country's densely populated areas, mainly the capital city Nairobi, the Lake Victoria, and the Coastal regions whilst the sparsely settled areas of north and eastern regions of the country are scarcely covered. Figure 2 shows the geographical locations of the health facilities that completed the survey. Of the total facilities (*n* = 219), the majority (61.6%; *n* = 135) offered no culture testing, 21.5% (*n* = 47) had cultures only i.e., no antimicrobial susceptibility testing and 16.9% (*n* = 37) performed antimicrobial susceptibility testing (Table 1). There were slightly more facilities from the private (55.3%; *n* = 121) relative to the public sector (44.8%; *n* = 98), whereas the representation between urban (49.3%; *n* = 108) and rural (50.7%; *n* = 111) areas was balanced. A notably higher proportion of facilities in rural areas (72.1%) lacked culture testing compared to those in urban areas (50.9%). Similarly, only 7.2% (*n* = 8) of the participating facilities in rural areas performed susceptibility tests compared to 26.9% (*n* = 29) of those in urban areas. Availability of susceptibility testing increased with advancing facility level from 0% in community health units and dispensaries to 100% in national referral hospitals. Further details on differences across laboratory affiliation, level, urbanicity, and administrative region are represented on Table 1.

**Table 1:** Health facilities completing the survey by affiliation, level, and urbanicity in regions of Kenya, 2020.

	**No culture testing (135; 61.6%)**	*Cultures only^d^ (47; 21.5%)	**AST (37; 16.7%)**	**All (*N* = 219)**
**Affiliation**, ***n*** **(%)**
*Public^a^	62 (63.3)	16 (16.3)	20 (20.4)	98 (44.7)
*Private^b^	73 (60.3)	31 (25.6)	17 (14.0)	121 (55.3)
**Level**, ***n*** **(%)**
Level 6 (National)	0 (0.0)	0 (0.0)	5 (100.0)	5 (2.3)
Level 5 (County referral)	6 (22.2)	6 (22.2)	15 (55.6)	27 (12.3)
Level 4 (Sub-County)	31 (45.6)	28 (41.2)	9 (13.2)	68 (31.1)
Level 3 (Health Centers)	26 (70.3)	10 (27.0)	1 (2.7)	37 (16.9)
Level 2 (Dispensaries)	36 (97.3)	1 (2.7)	0 (0.0)	37 (16.9)
Level 1 (Community)	30 (93.8)	2 (6.3)	0 (0.0)	32 (14.6)
Research	2 (33.3)	2 (33.3)	2 (33.3)	6 (2.7)
*Other^c^	3 (25.0)	2 (16.7)	7 (58.3)	12 (5.5)
**Urbanicity** ***n*** **(%)**
Urban	55 (50.9)	24 (22.2)	29 (26.9)	108 (49.3)
Rural	80 (72.1)	23 (20.7)	8 (7.2)	111 (50.7)
**Administrative region** ***n*** **(%)**
Central	16 (59.3)	5 (18.5)	6 (22.2)	27 (12.3)
Coast	10 (58.8)	3 (17.6)	4 (23.5)	17 (7.8)
Eastern	13 (44.8)	11 (37.9)	5 (17.2)	29 (13.2)
Nairobi	25 (52.1)	9 (18.8)	14 (29.2)	48 (21.9)
North Eastern	3 (100.0)	0 (0.0)	0 (0.0)	3 (1.4)
Nyanza	37 (72.5)	8 (15.7)	6 (11.8)	51 (23.3)
Rift Valley	12 (54.5)	9 (40.9)	1 (4.5)	22 (10.0)
Western	19 (86.4)	2 (9.1)	1 (4.5)	22 (10.0)

N, sample size;

a *Public includes government facilities and academic institutions.

b *Private includes entities supported by faith-based and non-government organizations as well as those run for profit by individuals or non-public companies.

c *Other include facilities of non-public ownership that do not fall in the indicated level categories.

d *Cultures only facilities offer bacterial culture services but no AST. AST, antimicrobial susceptibility testing.

### Strengths and gaps in quality assurance and management of data among AST facilities

Indicators to evaluate antimicrobial susceptibility testing facilities were distributed across 2 subdimensions: “quality assurance” and “management and dissemination of AMR data.”

**Figure 3:**
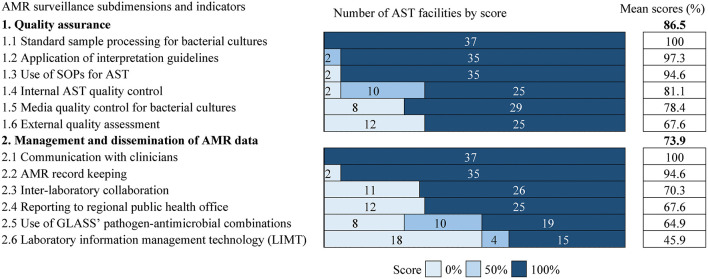
The figure provides information on the distribution of performance scores for AMR surveillance practices across facilities. The list of subdimensions and their component indicators is on the left. Shading and integers represent scores and facility count, respectively. The scores are ordered from left to right by increasing shade intensity. The number of facilities corresponds to column width. Percentage values on the right are the average score of all cells in each row. Average scores are ranked in descending order by subdimension and component indicator. AST, Antimicrobial Susceptibility Testing; SOPs, Standard operating procedures.

The AST facilities recorded an overall high performance (average score: 86.5%) in “quality assurance” with scores >80 % (facility is adequate) in four of six indicators (Figure 3). However, a substantial gap was identified in “external quality assessment” (score 67.6%) as 12 (32.4%) facilities reported non-participation in external quality assessment (EQA) programs for bacterial species isolation. Uptake of the EQA programs was generally balanced in facilities in rural and urban settings and those in public and private sectors (Supplementary File 2).

For the subdimension “management and dissemination of AMR data” (average score: 73.9%), facilities were strong in “communication with clinicians” (score: 100%) and “AMR record keeping” (score: 94.6%) but significantly weak in “laboratory information management technology” (LIMT) i.e., software to support systematic collation, analysis and sharing of microbiology data (score: 45.9%) (Figure 3). LIMT was particularly scarce in rural (25%) relative to urban (58.6%) areas but similarly available in the public- (35%) and private (47.1%) sectors (Supplementary File 2). The availability of LIMT also varied regionally, being available in more facilities in Nairobi (92.9%) followed by the Central (50%) administrative regions (Supplementary File 2). GLASS (Global Antimicrobial Resistance and Use Surveillance System) specified pathogen-antimicrobial combinations (26) were fully applied in about half of the facilities (score: 51.4%; *n* = 19) and partially applied in 10 (score: 13.5%) (Figure 3). Where GLASS guidelines were partially applied (*n* = 10), the list of antimicrobial agents provided by WHO and the priority pathogens for surveillance in Sub-Saharan Africa were modified.

### Comparison of infrastructural and resource capacities across study facilities

**Figure 4:**
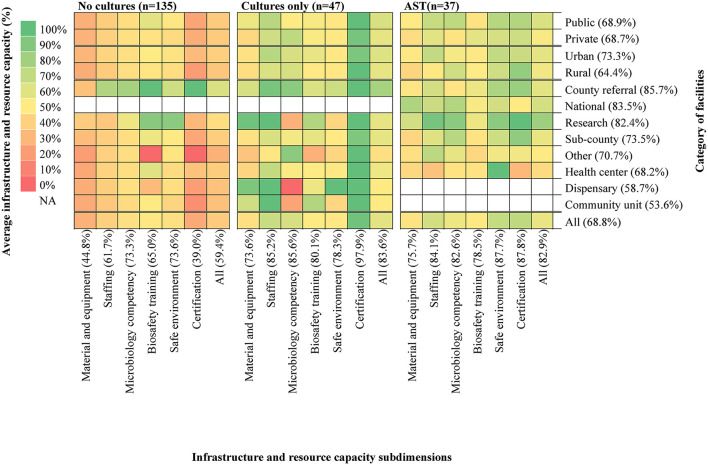
The heat map details infrastructure and resource capacity scores of the study facilities. The list to the right of the map indicates category names for facility affiliation (Private, Public) urbanicity (Rural, Urban) and level (National referral, Research, County referral, County, Health Centers, Dispensaries and Community units). The category “Other” includes facilities of non-public ownership that fall outside the 6-level structure of the Kenyan health system. The list below indicates the subdimensions of assessment for infrastructure and resource capacity. Indices in parentheses after each category name is the average capacity score of all cells in each row for left list and all cells in each column for list below. Categories are ranked in descending order of average capacity score for affiliation, urbanicity and level, respectively. AST, Antimicrobial Susceptibility Testing; NA, Not Applicable.

Health laboratories' infrastructure and resource capacities were evaluated in terms of “material and equipment,” “staffing,” “microbiology competency,” “biosafety training,” “safe environment,” and “certification” based on multiple indicators as detailed in Supplementary File 1. Generally, the laboratories demonstrated varied capacities across facility level and type (Figure 4). Community units and dispensaries required the most significant infrastructural strengthening (scores <60%) whereas county and national referral hospitals as well as research centers seemed to be performing well (scores > 80%). Across the three facility types investigated, those that lacked culture testing recorded the lowest average score (59.4%), with the subdimensions “material and equipment” (score 44.8%), and “certification” (score 39.0%) requiring significant strengthening. A total of 117 (53.4%) of all facilities were certified whereas 30 (13.7%) were in the process of receiving certification. Interestingly, in facilities where cultures only (average score: 83.6%) or AST (average score: 82.9%) were available, capacity scores were quite similar in all categories, with “certification,” “staffing,” and “microbiology competency” ranking the highest. Scores varied minimally across facilities in urban (73.3%) and rural (64.4%) areas, but were similar between the public (68.9%) and private (68.7%) sectors. Facilities had moderate to high scores in ‘safe environment' (73.6–87.7%) and “biosafety training” (65.0–80.1%) although 11% (*n* = 24) reported to never receiving any training in biosafety. The other 89 % (*n*= 195) receives the training between once in 2 years (*n* = 16) to twice a year (*n* = 100).

### Obstacles to antimicrobial susceptibility testing among culture-performing facilities

Several reasons for the inability to perform antimicrobial susceptibility testing among facilities with culture services were provided (Table 2). Unavailability of equipment was identified as the leading challenge to testing for resistance by 46.8% of the facilities, particularly those in the public sector (62.5%). Most of the laboratories (68%; *n* = 32) lacked −70°C freezers, followed by water distillation systems (38.3%; *n* = 18), blood culture machines (29.8%; *n* = 14), safety cabinet level 2 (23.4%; *n* = 11), atmosphere generating systems (23.4%; *n* = 11), glass or disposable petri dishes (21.3%; *n* = 10), warm air incubators (21.3%; *n* = 10), and manual pipettes (12.8%; *n* = 6). Besides, lack of funds (43.8%) and the acquisition and maintenance of supplies (56.5%) were cited as challenges for the public sector in comparison to the private sector. Inadequate competency among personnel was the least identified challenge across the facilities, at only 4.3%. Aside the outlined challenges, 46.8% of the facilities reported to refer samples to other facilities for susceptibility testing.

**Table 2:** Barriers hindering implementation of antimicrobial susceptibility testing among facilities with culture services in regions of Kenya.

	**All facilities**	**Public**	**Private**
**Barriers**	**(*n* = 47)**	**(*n* = 16)**	**(*n* = 31)**
Lack of equipment	22 (46.8)	10 (62.5)	12 (38.7)
Samples processed at partner facility	22 (46.8)	5 (31.3)	17 (54.8)
Lack of funds	14 (29.8)	7 (43.8)	7 (22.6)
Challenges obtaining supplies of reagents and materials	10 (21.3)	9 (56.5)	1 (3.2)
Lack of skilled personnel	2 (4.3)	0 (0)	2 (6.5)

n, sample size.

## Discussion

According to the present assessment, health facilities in multiple regions of Kenya require strengthening in key laboratory areas including, but not limited to, laboratory information management technology, external quality assurance, and material and equipment. In sub-Saharan Africa, robust information management structures to support AMR surveillance are limited. National AMR data systems are few and examples include the East Africa Public Health Laboratory Network (EAPHLN) sentinel site project (27) and Mapping Antimicrobial Resistance and Antimicrobial Use Partnership (MAAP), now covering 14 countries across West, East and Southern Africa (28). In high-income settings where well-functioning AMR surveillance systems exist, technologies for data management are integrated into most health systems as is in several European countries (29, 30). In such settings, inter-country benchmarking of AMR trends (30) is possible and reliable AMR information is available for action. Thus, bridging the technological gap in health facilities in Kenya could enhance effective analysis and output of credible results for clinical case management and policy use.

Access to laboratory technology was more than two-fold higher in facilities in urban relative to rural areas. This finding mirrors the longstanding maldistribution of health-care delivery common in low- and middle-income countries (31). Since disease burden entwined with drug regulatory problems are prominent in remote and poor areas (32), mitigating the inequitable access to laboratory technology is essential for improved representative AMR surveillance.

The study also identified a key gap in quality assurance, particularly low uptake of external quality assessment (EQA) programmes for bacterial species identification. Within Africa, WHO launched a regional microbiology EQA programme in 2002 that initially supported 39 national public laboratories from 30 member states. As of 2009, participating laboratories had doubled and 18 more member states had enrolled (33). Although this suggests that implementation of EQA programmes in Africa has improved over time, a vast majority of peripheral laboratories still lack EQA provision (34). In Kenya for instance, the WHO program serves two national facilities (35), a pattern that is likely to be similar throughout sub-Saharan African countries. There is therefore a need for the establishment of effective EQA schemes for bacterial identification and antimicrobial susceptibility testing in developing countries in order to ensure accuracy of laboratory investigations.

Poor internal quality control mechanisms were found among the participating facilities. This was evident in the limited use of control strains for cultures in several facilities, posing a challenge over the credibility of results generated by the laboratories. Whereas, the use of unified international guidelines (CLSI or EUCAST) for interpretation of susceptibility results was noted in almost all facilities, the application of WHO specified pathogen-antimicrobial combinations was infrequent or partial in some cases, which could undermine uniformity and comparability of AMR data on multiple levels.

Infrastructure and resource capacity was rather weak among laboratories that lacked culture testing, particularly health centers, dispensaries, and community units. Addressing the inadequacies would be of great benefit to an estimated 36% of Kenya's population (36), comprising the vast rural population primarily served by these facilities (37). Notably, laboratories with cultures only and those with AST showed similar strengths in capacities. These findings hint at a potential target opportunity of upgrading facilities that perform cultures to implement antimicrobial susceptibility testing, with minimum investment. Such investments through the national and county governments in collaboration with development partners would greatly improve healthcare provision as well as AMR surveillance. Obstacles to the implementation of AST were lack of equipment and funding, while trained personnel seemed to be available. With the existing infrastructure and trained workforce in place, we suggest that future healthcare projects prioritize investment and procurement of new low-maintenance and easy to repair equipment to help enhance overall laboratory capacity. Moreover, upgrading facilities could help circumvent transport costs and reduce turnaround time for facilities that send out samples to external laboratories for testing. Our findings highlight that facilities in the private sector did not face significant challenges in obtaining and maintaining supply of reagents and materials, yet more than half of those in the public sector cited this problem. This finding suggests that supplies can be obtained in Kenya, although it also exposes potential procurement obstacles in the public sector. Therefore, revisiting laboratories in this sector to identify supply constraints and institute corrective measures is recommended.

The study has some limitations beginning with that it was not designed to investigate the capacity of laboratories to confirm and interpret unexpected phenotypes. Secondly, data were self-reported, a limitation brought about by strict COVID-19 restrictions that prevented on-site visits and minimized independent survey verification. Also, binary responses may lead to overly optimistic assessments with regards to true capacity and true performance. Finally, the generalizability of the current findings is limited as some geographic regions are barely represented among the facilities that participated in the study. Since the data was collected *via* a web-based program, limited internet access, unreliability of email addresses, and lack of electronic appliances may have contributed to the disproportionate representation.

Although not all geographical areas are covered, the survey includes health facilities in very diverse settings of Kenya; from rural to urban sites, from Lake Victoria to the Indian Ocean, providing a good reflection of the country's laboratory capacity status. In resource limited settings, strengthening of health facilities require effective planning toward achieving universal coverage. It is therefore important to note that all clinical laboratories offering some microbiology services, especially microscopy, need not be able to provide culture and susceptibility testing capabilities. Ideally all geographic regions and patients should have access to culture and susceptibility tests, but not necessarily within each laboratory facility.

## Conclusion

We effectively applied a quantitative evaluation among health laboratories in multiple regions of Kenya and found gaps in information management technology, external quality assurance, and material and equipment. Our findings suggest that by investing in equipment, facilities performing cultures can be successfully upgraded to provide additional antimicrobial susceptibility testing, presenting a chance for a major leap forward toward improved AMR diagnostics and surveillance in the country. Based on the gaps identified, we recommend increased access to laboratory information management technology for enhanced AMR data management and communication. As a national commitment, targeted quality assurance mechanisms for microbiology facilities are likely to greatly improve overall healthcare delivery. Also, long-term financing mechanisms are needed to improve testing capacity particularly at health center, dispensary and community facility levels where infrastructural deficiencies were most notable. In essence, our findings can serve as a basis to gauge the impact of these interventions and the scoring tool developed for the study could be applied in comparable gap contexts. Moreover, the evaluation tool applied in this study can be used by facilities to independently assess their infrastructure and resource capacities and evaluate their practices.

## Data availability statement

The raw data supporting the conclusions of this article will be made available by the authors, without undue reservation.

## Author contributions

RM, DE, and EL contributed toward conceptualization and study design. RM, BA, and SA conducted the survey. RM processed, analyzed, and interpreted data and wrote the first draft of the paper. DE and EL supervised study and contributed toward data interpretation. DE, LA, and EL supported the writing. SY took part in editing. JM and ML contributed toward funding acquisition. All authors read and approved the final manuscript.

## Funding

This study was made possible through a grant from the German Federal Ministry of Health (BMG) through the Global Health Protection Program (GHPP) (Grant No. ZMV15 2519 GHP 705).

## Conflict of interest

The authors declare that the research was conducted in the absence of any commercial or financial relationships that could be construed as a potential conflict of interest.

## Publisher's note

All claims expressed in this article are solely those of the authors and do not necessarily represent those of their affiliated organizations, or those of the publisher, the editors and the reviewers. Any product that may be evaluated in this article, or claim that may be made by its manufacturer, is not guaranteed or endorsed by the publisher.

## Footnotes

^1^ https://apps.who.int/iris/handle/10665/204423

^2^ https://roggkenya.org/2019/07/22/kenyas-health-structure-and-the-six-levels-of-hospitals-an-overview/

^3^ https://redcapinfo.ucdenver.edu/citing-redcap.html

## Abbreviations

AMR: Antimicrobial Resistance
AST: Antimicrobial Susceptibility Testing
ATCC: American Type Culture Collection
CDC: Centers for Disease Control and Prevention
COVID-19: Coronavirus Disease
CLSI: Clinical and Laboratory Standards Institute
CDDEP: Center for Disease Dynamics Economics & Policy
EA-REQAS: East African Regional External Quality Assessment Scheme
EAPHLN: East Africa Public Health Laboratory Networking
EQA: External Quality Assessment
EUCAST: The European Committee on Antimicrobial Susceptibility Testing
GHPP: Global Health Protection Program
GLASS: Global Antimicrobial Resistance and Use Surveillance System
KIPPRA: Kenya Institute for Public Policy Research and Analysis
LIMT: Laboratory information management technology
MAAP: Mapping Antimicrobial Resistance and Antimicrobial Use Partnership
MOH: Ministry of Health
NACOSTI, National Commission for Science, Technology: and Innovation
NCTC: National Collection of Type Cultures
REDCap: Research Electronic Data Capture
SOPs: Standard Operating Procedures
SLIPTA: Stepwise Laboratory Improvement Process Towards Accreditation
WHO: World Health Organization.
