# Supplementary material for: Laboratory-based surveillance of antimicrobial resistance in regions of Kenya: An assessment of capacities, practices, and barriers by means of multi-facility survey
Source: Front Public Health. 2022 Nov 28;10:1003178. doi: 10.3389/fpubh.2022.1003178 (PMC9742437; doi:10.3389/fpubh.2022.1003178)
Supplement: Supplementary file 1 [file Table_1.docx]

Supplementary file 1 Scoring scheme developed for assessment of health facilities in Kenya, 2020

| **Dimension** | **Subdimension and Indicator** | **Question frame** | **Score** | **Weighting value** |
| --- | --- | --- | --- | --- |
| **1. AMR surveillance practices** | **1.1 Quality assurance** |  |  |  |
|  | 1. Media quality control | -use of specific bacterial control strains for culture | No=0, yes=1 | 1 |
|  | 1. Standard sample processing | -utilization of standard internal operating procedures for processing of samples for bacterial culture | No=0, yes=1 | 1 |
|  | 1. External quality assurance | -participation in external quality assessment system for isolation of bacteria | No=0, yes=1 | 1 |
|  | 1. Standardized antimicrobial susceptibility testing | -use of internal standard operating procedures for assuring the quality of ASTs | No=0, yes= 1 | 1 |
|  | 1. Internal AST quality control | -Use of ATCC/NCTC or other reference strains | No=0, other= 0.5, yes = 1 | 1 |
|  | 1. Application of international interpretation guidelines | -Use of CLSI/ EUCAST or other | No=0, other= 0.5, yes = 1 | 1 |
|  |  |  |  |  |
|  | **1.2 Management and dissemination of AMR data** |  |  |  |
|  | 1. Communication with clinicians | - AST results reported to healthcare provider | No= 0, yes=1 | 1 |
|  | 1. AMR record keeping | -AST records kept longterm | No=0, yes= 1 | 1 |
|  | 1. Inter-laboratory collaboration | - Information exchange between laboratories | No=0, yes= 1 | 1 |
|  | 1. Reporting to regional public health office | - Regular data submission to regional health office | No=0, yes= 1 | 1 |
|  | 1. Application of GLASS guidelines | -AST performed according to GLASS pathogen-antimicrobial combination | No=0, partial=0.5, yes=1 | 1 |
|  | 1. Laboratory information management technology | - Use of data management software such as WHONET. Other refers to local databases with no exchange of information | No=0, other =0.5, yes=1 | 1 |
|  |  |  |  |  |
| **2. Infrastructure & resource capacity** | 2. 1 Materials & Equipment |  |  |  |
|  | Category 1 | -Availability and function | Present and functional=1, present and non-functional=0.5, absent =0 | 1 |
|  | 1. Adequate glassware for media preparation (flasks, cylinders, etc)  2. Atmosphere generating systems or CO2 tanks and CO2 incubator or candle jars  3. Autoclave (manual or electrically controlled)  4. Blood culture machine  5. Bunsen burner or heater or lamp to sterilize loops and needles  6. Disposable loop/needle handles or Loop/needle handles or 0.01 and 0.001ml calibrated loops  7. -70°C Freezer  8. Manual pipettes (e.g Eppendorf)  9. Microscope with oil-immersion objective  10. Petri dishes (glass or disposable)  11. Refrigerator  12. Safety cabinet- level 2 (protects operator and material from contamination)  13. Scale or balance  14. Slides  15. Staining facilities- sink and slide rack  16. Warm air incubator  17. Water distillation system  18. Other anaerobe jar |  |  |  |
|  | The question frame, score and weighting value in category 1 applies to indicators 1-18. The response type ‘non-functional’ is not applicable for indicator 1, 6, 10, 14, 18. | | | |
|  | Category 2 | -Availability and function | Present and functional=1, present and non-functional=0.5, absent =0 | 0.8 |
|  | 1.Test tube racks  2.Vortex Mixer  3.Magnifying lens  4.Safety cabinet-level 3 (protects operator, material, and environment) |  |  |  |
|  |  |  |  |  |
|  | The question frame, score and weighting value in category 2 applies to indicators 1-4. The response type ‘non-functional’ is not applicable for indicator 1, 3. | | | |
|  | Category 3 | -Availability and function | Present and functional=1, present and non-functional=0.5, absent =0 | 0.6 |
|  | 1. Colorimeter  2. Electrically powered water bath  3. -20°C Freezer  4. Hot air oven  5. Inverted microscope  6. Low speed centrifuge (hand or electrically powered)  7. Safety cabinet- level 1 (protects material from contamination) |  |  |  |
|  |  |  |  |  |
|  | The question frame, score and weighting value in category 3 applies to indicators 1-7. The response type ‘non-functional’ is not applicable for indicator 1. | | | |
|  | Category 4 | -Availability and function | Present and functional=1, present and non-functional=0.5, absent =0 | 0.4 |
|  | 1. Coverslips  2. Multipoint inoculator  3. pH meter  4. pH paper  5. Fluorescent microscope |  |  |  |
|  |  |  |  |  |
|  | The question frame, score and weighting value in category 4 applies to indicators 1-5. | | | |
|  | **2.2 Staffing** | - On staff | Medical supervisor, No=0, yes=1 | 0.5 |
|  |  |  | Technical supervisor, No=0, yes=1 | 1 |
|  |  |  | Lab technologist, No=0, yes=1 | 1 |
|  |  |  | Lab assistant, No=0, yes=1 | 0.5 |
|  |  |  | Epidemiologist, No=0, yes=1 | 0.45 |
|  |  |  | Microbiologist, No=0, yes=1 | 1 |
|  |  |  | Clerical staff, No=0, yes=1 | 0.25 |
|  |  |  | Other e.g supportive staff, No=0, yes=1 | 0.2 |
|  |  |  |  |  |
|  | **2.3 Microbiology competency** | - Level of microbiology training | Master’s degree= 1, Bachelor’s degree= 0.9, Diploma course=0.75, In-house training= 0.5, None=0 | 1 |
|  |  |  |  |  |
|  | **2.4 Safety training** | -Frequency of training on laboratory safety | >twice a year =1, twice a year= 0.9, once a year =0.75, once in two years =0.5, never=0 | 1 |
|  |  |  |  |  |
|  | **2.5 Safe practices in safe environment** | -Presence of personal protective items (e.g Lab coats, gloves, visors) | None =0 | 0 |
|  |  |  | only visors = 0.2 | 0.2 |
|  |  |  | only lab coat = 0.35, | 0.35 |
|  |  |  | only gloves = 0.35, | 0.35 |
|  |  |  | only other =0.15, | 0.15 |
|  |  |  | only other and visors =0.25, | 0.25 |
|  |  |  | only visors and lab coat = 0.6, | 0.6 |
|  |  |  | only visors and gloves = 0.6, | 0.6 |
|  |  |  | only gloves and lab coats = 0.7, | 0.7 |
|  |  |  | only other and gloves = 0.5, | 0.5 |
|  |  |  | only other and lab coat = 0.5, | 0.5 |
|  |  |  | only other, gloves and visors =0.8, only | 0.8 |
|  |  |  | other, lab coat and visors =0.8, only | 0.8 |
|  |  |  | other, gloves and lab coat = 0.85, | 0.85 |
|  |  |  | only visors, lab coat and gloves =0.9 | 0.9 |
|  |  |  | Lab coat, gloves, visors and other =1 | 1 |
|  |  |  |  |  |
|  | - 1. **Certification** | -Confirmation of accreditation | No accreditation=0, Accreditation in progress= 0.5, accredited= 1 | 1 |

AST, Antimicrobial Susceptibility Testing; GLASS, Global Antimicrobial Resistance and Use Surveillance System; LIMT, Laboratory information management technology; SOPs, Standard operating procedures; ATCC, American Type Culture Collection; NCTC, National Collection of Type Cultures; CLSI, Clinical and Laboratory Standards Institute; EUCAST, European Committee on Antimicrobial Susceptibility Testing.
