# Supplementary material for: Laboratory-based surveillance of antimicrobial resistance in regions of Kenya: An assessment of capacities, practices, and barriers by means of multi-facility survey
Source: Front Public Health. 2022 Nov 28;10:1003178. doi: 10.3389/fpubh.2022.1003178 (PMC9742437; doi:10.3389/fpubh.2022.1003178)
Supplement: Supplementary file 2 [file Table_2.docx]

**Supplementary file 2 Proportion of antimicrobial testing facilities in regions of Kenya by gap identified, affiliation, level, urbanicity**

|  | **LIMT availability (15;40.5%)** | **EQA participation (25;67.6%)** | **Regional level reporting (25;67.6%)** | **GLASS pathogen-antimicrobial combinations (19;51.4%)** | **All (N=37)** |
| --- | --- | --- | --- | --- | --- |
| **Affiliation (%)** |  |  |  |  |  |
| *Public^1^ | 7(35.0) | 13(65.0) | 14(70.0) | 9(45.0) | 20 |
| *Private^2^ | 8(47.1) | 12(70.6) | 11(64.7) | 10(58.8) | 17 |
| **Level (%)** |  |  |  |  |  |
| National referral | 5(100.0) | 5(100.0) | 4(80.0) | 5(100.0) | 5 |
| Research | 2(100.0) | 1(50.0) | 2(100.0) | 2(100.0) | 2 |
| County referral | 6(40.0) | 8(53.3) | 9(60.0) | 5(33.3) | 15 |
| Sub-county | 1(11.1) | 8(88.9) | 6(66.7) | 3(33.3) | 9 |
| Health centres | 0(0.0) | 0(0.0) | 1(100.0) | 0(0.0) | 1 |
| *Other^3^ | 3(42.9) | 5(83.3) | 5(71.4) | 6(85.7) | 7 |
| **Urbanicity (%)** |  |  |  |  |  |
| Rural | 2(25.0) | 6(75.0) | 5(62.5) | 2(25.0) | 8 |
| Urban | 13(58.6) | 19(65.5) | 20(69.0) | 17(58.6) | 29 |
| **Administrative region (%)** |  |  |  |  |  |
| Central | 3(50.0) | 5(83.3) | 5(83.3) | 1(16.7) | 6 |
| Coast | 0(0.0) | 3(75.0) | 2(50.0) | 2(50.0) | 4 |
| Eastern | 1(20.0) | 4(80.0) | 2(40.0) | 2(40.0) | 5 |
| Nairobi | 13(92.9) | 11(78.6) | 10(71.4) | 11(78.6) | 14 |
| Northeastern | NA | NA | NA | NA | 0 |
| Nyanza | 2(33.3) | 2(33.3) | 6(100.0) | 3(50.0) | 6 |
| Rift Valley | 0(0.0) | 0(0.0) | 0(0.0) | 0(0.0) | 1 |
| Western | 0(0.0) | 0(0.0) | 0(0.0) | 0(0.0) | 1 |

N, sample size; ^1^*Public includes government facilities and academic institutions. ^2^*Private includes entities supported by faith-based and non-government organizations as well as those run for profit by individuals or non-public companies. ^3^*Other include facilities of non-public ownership that fall outside the indicated level categories. LIMT, Laboratory information management technology; EQA, External quality assessment; GLASS, Global Antimicrobial Resistance and Use Surveillance System
